# Ready for SDM: evaluating a train-the-trainer program to facilitate implementation of SDM training in Norway

**DOI:** 10.1186/s12911-021-01494-x

**Published:** 2021-04-30

**Authors:** Simone Kienlin, Marie-Eve Poitras, Dawn Stacey, Kari Nytrøen, Jürgen Kasper

**Affiliations:** 1 Department of Health and Caring Sciences, Faculty of Health Sciences, University of Tromsø, Postbox 6050, Langnes, Norway; 2 Division of Internal Medicine, University Hospital of North Norway, Postbox 100, 9038 Tromsø, Norway; 3 Department of Medicine and Healthcare, The South-Eastern Norway Regional Health Authority, Postbox 404, 2303 Hamar, Norway; 4 Department of Family Medicine and Emergency Medicine/School of Nursing, Faculty of Medicine and Health Sciences, Université de Sherbrooke, Sherbrooke, Canada; 5 School of Nursing, University of Ottawa, 451 Smyth Road, Ottawa, ON K1H 8M5 Canada; 6 Ottawa Hospital Research Institute, 501 Smyth Road, Ottawa, ON K1H 8L6 Canada; 7 Faculty of Medicine, University of Oslo, Blindern, Postbox 1072, 0316 Oslo, Norway; 8 Department of Nursing and Health Promotion, Faculty of Health Sciences, OsloMet Metropolitan University, Pilestredet 46, 0167 Oslo, Norway

**Keywords:** Shared decision-making, Train-the-trainer, Curriculum, Communication skills, Education, Complex intervention

## Abstract

**Background:**

Healthcare providers need training to implement shared decision making (SDM). In Norway, we developed “Ready for SDM”, a comprehensive SDM curriculum tailored to various healthcare providers, settings, and competence levels, including a course targeting interprofessional healthcare teams. The overall aim was to evaluate a train-the-trainer (TTT) program for healthcare providers wanting to offer this course within their hospital trust.

**Methods:**

Our observational descriptive design was informed by Kirkpatrick´s Model of Educational Outcomes. The South-Eastern Regional Health Authority invited healthcare providers from all health trusts in its jurisdiction to attend. The TTT consisted of a one-day basic course with lectures on SDM, exercises and group reflections followed by a two-day advanced course including an SDM observer training. Immediately after each of the two courses, reaction and learning (Kirkpatrick levels 1 and 2) were assessed using a self-administered questionnaire. After the advanced course, observer skills were operationalized as accuracy of the participants’ assessment of a consultation compared to an expert assessment. Within three months post-training, we measured number of trainings conducted and number of healthcare providers trained (Kirkpatrick level 3) using an online survey. Qualitative and quantitative descriptive analysis were performed.

**Results:**

Twenty-one out of 24 (basic) and 19 out of 22 (advanced) healthcare providers in 9 health trusts consented to participate. The basic course was evaluated as highly acceptable, the advanced course as complex and challenging. Participants identified a need for more training in pedagogical skills and support for planning implementation of SDM-training. Participants achieved high knowledge scores and were positive about being an SDM trainer. Observer skills regarding patient involvement in decision-making were excellent (mean of weighted t = .80). After three months, 67% of TTT participants had conducted more than two trainings each and trained a total of 458 healthcare providers.

**Conclusion:**

Findings suggest that the TTT is a feasible approach for supporting large-scale training in SDM. Our study informed us about how to improve the advanced course. Further research shall investigate the efficacy of the training in the context of a comprehensive multifaceted strategy for implementing SDM in clinical practice.

*Trial registration*: Retrospectively registered at ISRCTN (99432465) March 25, 2020.

**Supplementary Information:**

The online version contains supplementary material available at 10.1186/s12911-021-01494-x.

## Background

Patient participation in healthcare decisions is an essential element of evidence-based medicine and patient-centered care [1]. Despite strong international advocacy for SDM and increasing implementation efforts, it has not yet been routinely adopted in clinical practice [2, 3]. Numerous interventions exist to support patients and clinicians in the process of making decisions using the best available evidence and the patient’s informed preferences [4]. Many involve interventions targeting patients, such as patient decision aids and decision coaching, and interventions targeting healthcare providers (HCPs) to increase patient involvement, such as training programs. Evidence on the efficacy of SDM training programs, however, is poor. This applies with regard to a lack of transparency in the reporting of training methods used, heterogeneity across descriptions of SDM training programs, and a lack of training programs which are rigorously evaluated [5, 6].

In Norway, there is growing interest in implementing SDM. The Norwegian Ministry of Health has published a series of documents indicating the need for more SDM [7–10]. The latest contribution is the National Health and Hospital Plan 2020–2023 [10], which considers SDM as a key goal and best practice for making healthcare decisions. Several university health and social education programs, including medical specialization programs, have recently been given specific SDM learning objectives [11, 12]. In Norway, the meta curriculum “Ready for SDM” (in Norwegian, *Klar for Samvalg*) is recommended as one strategy to support implementation of SDM.  The meta-curriculum consists of several SDM training modules using both classroom and online format—and guidance for tailoring SDM training to the different contexts and needs of HCPs. The curriculum is based on MAPPIN´SDM (Multifocal Approach to the Sharing in SDM) as its underpinning concept of SDM quality [13, 14]. MAPPIN´SDM is an inventory hosting several instruments to assess patient involvement and a research approach to compare and integrate varying perspectives on the quality of decision-making communication. MAPPIN´SDM defines the chronological steps of an SDM approach and provides detailed descriptions of several levels of performance for each quality indicator [15, 16]. MAPPIN’SDM is founded on the criteria of evidence-based patient information [15, 16] and, in a recent systematic review comparing measurement instruments, has been found to most comprehensively cover the essential elements of SDM [17]. Using a generic pedagogic approach [18], the Ready for SDM meta-curriculum also relies on a set of “active components” used to change behaviour of HCPs, so-called behaviour change techniques (BCTs) [19]. BCTs are observable and replicable and can be used individually or in combination with other BCTs from a comprehensive taxonomy of 93 techniques [19]. This evidence-based taxonomy is supposed to support creation of theory-informed implementation interventions [19] and may improve the evidence on SDM-trainings by aiding in transparent reporting of interventions [20].

The individual training modules within our meta-curriculum Ready for SDM currently present in different stages of evaluation [18, 21–24].

One module of our meta-curriculum, Ready for SDM INTERPROF, is using an interprofessional approach to facilitate translation of SDM into practice by improving knowledge and attitudes and thereby, changing the culture of communication in health care environments. This interactive 2-h classroom educational module has passed extensive qualitative evaluation [18] and in a cluster randomized study proved efficacious regarding knowledge gain and acquisition of communication competencies [25]. It is currently in frequent demand by hospital trusts, is recommended by the Ministry of Health, and may soon play an important role in Norwegian national strategies to implement SDM in health care [18, 25]. However, to scale up SDM activities in hospitals, transition to disseminating interprofessional SDM training through a train-the-trainer program (TTT) is required. Such an approach needs to ensure fidelity of the intervention as originally designed.

The overarching goal of this study was to scale up SDM training for health professionals in Norwegian hospital trusts by evaluating a train-the-trainer program. Specifically, we aimed to evaluate the extent to which our TTT program gives HCPs, as ambassadors, the skills and confidence to provide the Ready for SDM INTERPROF module for groups of HCPs in their respective practice environments.

## Methods

### Study design

We pretested the TTT in a group of HCPs using an observational descriptive design with evaluation based on the Kirkpatrick model [27]. Kirkpatrick’s model of outcomes for evaluating educational interventions consists of four levels: Level 1 assesses immediate response; Level 2 assesses learning effects such as knowledge, skills, and attitudes; Level 3 assesses behavioural change due to the training, and Level 4 assesses the efficacy of the training. Evaluation in the current study refers to levels 1–3.

To describe the TTT intervention, we used the Template for Intervention Description and Replication (TIDieR) checklist [28], increasingly used to describe complex interventions [29, 30].

### Intervention description

#### Rationale

The goal of the TTT program was to prepare HCPs to provide the Ready for SDM-INTERPROF module to their colleagues. Besides handing over the necessary learning materials and presentation slides, TTT trainers teach participants how to implement the 2-h curriculum themselves. Participants also become introduced to the underpinning concept of quality, MAPPIN’SDM, and learn to apply the corresponding quality criteria to appraisal of communication.

As one piece of a more comprehensive implementation approach of SDM in Norway, the TTT was developed in accord with the Knowledge-to-Action framework (KTA) [31]. The framework guides implementation endeavors via seven abstract steps (Table 1). The current study contributes to covering each of the KTA steps. In addition, it refers to many specific interventions completing the overarching approach of the South-Eastern Health Region (see Table 1).

**Table 1:** Knowledge-to-Action plan for Ready for SDM INTERPROF

Stages of KTA	*Reported elsewhere/*Reported in current study
(1) Identify the gap	*SDM and SDM trainings are not yet implemented in clinical practice in Norway* *Until recently, there have been no SDM learning goals in social and medical training* *76% of doctors in medical residency training reported having had no SDM training (n* = *111)* *Low levels of patient involvement in Norwegian specialist health care have been found* [14] *SDM training in Norway has only just begun* [18, 25, 26] SDM INTERPROF has proven efficacious. Its distribution requires the use of a TTT module
(2) Adapt knowledge to local context	The TTT curriculum enables ambassadors to adapt SDM INTERPROF to their local needs In pilots and pretests of SDM INTERPROF the curriculum has been adapted to several local medical contexts [18, 25] The target group participated in developing SDM INTERPROF in the context of a quality improvement project
(3) Access barriers to knowledge use	*Knowledge about barriers:* *Evidence of barriers to SDM implementation in literature* [51–53] *Barriers assessed in our previous studies:* *1-Pretest of SDM INTERPROF* [18] *2-RCT on efficacy of SDM INTERPROF* [25] Barriers collected during piloting of the TTT module and a focus group study
(4) Select, tailor, implement interventions	*SDM INTERPROF showed it was feasible and efficacious* TTT selected as a strategy for more efficient and tailored implementation of SDM INTERPROF
(5) Monitor knowledge use	Post intervention survey assessed further redistribution of SDM INTERPROF by participants A quality collaborative will share and discuss experiences Workshop 12 months post-TTT with participants to assess experiences of applying SDM INTERPROF, including barriers to sustainability
(6) Evaluate outcomes	Level 1: Engagement, relevance and satisfaction assessed Level 2: Knowledge, skills, confidence and commitment assessed Level 3: A reporting system established to monitor number of trainings delivered and trainees trained *Level 4: Measure SDM in clinical practice using MAPPIN´SDM for a select group of patients in the South-Eastern Regional Health Authority*
(7) Sustain knowledge use	Conducting new TTT courses as new staff is hired *In preparation:* *Develop and assess feasibility of additional SDM training modules* *Establish a support system for SDM ambassadors* *Revise and update the klarforsamvalg.no* [34] *homepage*

This table illustrates how the seven stages of the KTA framework [31] guide the systematic implementation of SDM trainings in healthcare. Italic text refers to parts of the overall Ready for SDM strategy reported elsewhere or planned for the future. “Ambassadors” is the term used for HCPs certified as trainers. “Levels” under stage [6] refers to Kirkpatrick’s evaluation levels [27]. “MAPPIN’SDM” under stage [6] is a validated measurement instrument to assess the extent of patient involvement in consultations [14]

#### Description of the SDM intervention to be passed on: ready for SDM INTERPROF

The Ready for SDM INTERPROF [18] is a two-hour group-based interprofessional module, training HCPs‘ SDM knowledge and skills aiming at facilitating a culture change in HCP-patient communication. It was developed following the assumption that to work as a convincing communication approach, patient involvement needs to be reflected by shared attitudes amongst clinical teams. SDM skills do therefore not necessarily refer to decision-making consultations only, but also to all communication pieces involved in providing information and preparing patients for taking an active role on their own account. Using a lecture and interactive methods the INTERPROF module addresses the particular learning goals: To gain knowledge on background and rationale of SDM and risk communication, skills to structure an SDM process using “6 steps to SDM” and develop self-appraisal skills using quality criteria from the MAPPIN’SDM [18, 25].

#### Development of the train-the-trainer curriculum

The main learning objective of the TTT course is to build competence and confidence among participants who will provide the Ready for SDM INTERPROF module to further groups of HCPs. The TTT curriculum was developed by an expert panel consisting of a patient representative, a web editor and communication specialist, and researchers with clinical, educational and leadership expertise.

Using “blended learning” and adult learning approaches [32] as well as strategies from the Ready for SDM meta-curriculum [18, 21], the TTT includes presentations, group discussions, exercises, interactive observation and demonstration**.** Pedagogic methods were selected that were appropriate for bigger groups and still keeping focus on interactivity in the learning. The course was designed to help trainers address known barriers to SDM (such as the belief that HCPs are already doing SDM, or that their patients don’t want to share decisions) and also to help them identify others [19, 20]. Allocating our methods in the taxonomy and considering use of additional BCTs helped us refining the curriculum and to make it traceable for other educators and researchers (Table 2) [19, 20]. For example, one BCT used to address the barrier that HCPs already do SDM is “use of a credible source,” i.e. presenting evidence on average level of patient involvement. Beyond the use of specific BCTs and their operationalization, when a barrier was mentioned during the training, it was met using a generic sequence: First it was rephrased a couple of times to enable other participants to recognize its relevance to their own situation. Then trainers affirmed the barrier mentioned by the trainee before specific information and arguments were provided to address the barrier (Table 2).

**Table 2:** Barriers to implementation of SDM and BCTs used to address them in the TTT training

Beliefs/concerns/attitudes that constitute the barrier	Attributed by whom	How the barrier affects implementation	Relevant BCT to address the barrier	Operationalization of BCT in TTT training
Patients do not want to participate in making decisions	HCP & trainer	Patient involvement is not considered	Use of a credible source (9.1)	Evidence about patients’ preferences about taking control of their health choices and about HCPs’ flawed assumptions about what patients want are provided in Powerpoint presentation
			Information about social and environmental consequences (5.3)	The training refers to national and regional policies and ethical guidelines supporting SDM
			Providing prompts /cues (7.1)	Materials are shared: Patient activation brochure and poster, 6 steps to SDM “pocket reminder cards”
We are already doing SDM	HCP & trainer	Potential for improvement in patient involvement	Use of a credible source (9.1)	Evidence is provided on average level of patient involvement by HCPs
			Instruction on how to perform the behaviour (4.1)	A structure for decision-making that involves patients is suggested using: 6 steps to SDM “pocket reminder cards” and example videos
			Feedback on behaviour (2.2)	Feedback is provided to a model (HCP presented in a video example)
			Demonstration of the behaviour (6.1)	The suggested consultation structure, 6 steps to SDM, is demonstrated using video examples
			Social comparison (6.2)	HCP opinion-leaders are presented using video examples
Trainer feels insufficiently supported by management	Trainer	HCP – SDM trainings will not lead to behaviour change	Provision of/enabling social support (3.1–3)	Encouragement to make use of a permanent supervision offer to receive counselling communication on implementation of SDM at the hospital trusts
			Information about social and environmental consequences (5.3)	The training refers to national and regional policies and ethical guidelines to implement SDM
SDM takes too much time	HCP & trainer	Patient involvement is not considered or essential elements are omitted	Use of a credible source (9.1)	Evidence is presented challenging the claim that SDM is too time consuming
			Instruction on how to perform the behaviour (4.1) Demonstration of the behaviour (6.1)	A structure for consultations involving patients is suggested using: 6 steps to SDM “pocket reminder cards” and example videos
			Adding objects to the environment (12.5)	Trainees are introduced to Patient Decision Aids which have been developed to prepare patients for making health choices
An overarching implementation strategy is absent	Trainer	Ad hoc trainings might be carried out, but SDM is not implemented in a sustained fashion	Provision of/enabling social support (3.1–3)	Offer of assistance with implementation at their hospital trust
SDM is not relevant to us	HCP & trainer	Lack of awareness of preference sensitive decisions. HCPs might make decisions based on guidelines on behalf of the patients	Provision of/enabling social support (3.1–3)	During the training, the ambassadors are invited to a interprofessional network of SDM trainers and access to the “klarforsamvalg” webpage, hosting learning materials used in trainings is provided
			Information about social and environmental consequences (5.3)	The training refers to national and regional policies and ethical guidelines supporting SDM
			Information about health consequences (5.1)	Information is provided about effects of SDM on patient outcomes
Challenging to find the evidence for every medical problem	HCP & trainer	Decisions are not informed by best available evidence	Tailoring (Agbadjé 2020)*	Domain-specific decisions are identified using exercises
				Criteria for evidence-based patient information are introduced
			Adding objects to the environment (12.5)	Attention is called to Patient Decision Aids that are freely available on various health platforms
SDM is only about the doctors and their patients	HCP & trainer	Patient involvement might happen in isolated events (eg consultations), but is not implemented as a team culture	Tailoring (Agbadjé 2020) * Problem solving (1.1)	Exercises and group discussion are used to draft solutions to interprofessional role distribution regarding typical domain-specific decision scenarios
			Instruction on how to perform the behaviour (4.1)	Nurse-led decision coaching is presented using a sequence of PP slides as an example for interprofessional SDM
			Information about social and environmental consequences (5.3)	Emphasis is given to virtues of interprofessional cooperation: confidence, respect, appreciation, sharing competences
			Restructuring the social environment (12.2)	Advice to restructure information flow and interprofessional collaboration to promote patient involvement
Patients do not understand this information	HCP & trainer	HCP avoid providing evidence-based information	Use of a credible source (9.1)	Evidence is presented about patients’ ability to process evidence-based information
			Instruction on how to perform the behaviour (4.1)	The criteria for evidence-based patient information are introduced and reference is made to the guideline evidence-based health information
			Adding objects to the environment (12.5)	The trainers’ attention is called to tools and methods of risk communication
The trainer lacks opportunities to deliver the training	Trainer	SDM INTERPROF will not be implemented	Adding objects to the environment (12.5)	Trainers are equipped by materials for distribution and information to leaders
			Guidance of action planning (1.4)	Opportunities are provided in structured exercises to make plans on by whom, where and when SDM trainings will be carried out
Ambassador does not feel sufficiently confident as a trainer	Trainer	SDM training is not effective or does not comply with Ready for SDM	Provision of/enabling social support (3.1–3)	A didactic model for planning the training is provided and assistance offered to conduct SDM trainings locally
			Guidance of action planning (1.4)	Opportunities are provided in structured exercises to make plans about where and when SDM trainings will be carried out and by whom

Examples of barriers trainers or health care providers (HCP) meet when trying to implement SDM (shared decision making) and use of behavior change techniques (BCT) to address these barriers in the training. The generic techniques to meet barriers (acknowledging, rephrasing, information, argument and cognitive restructuring), are not specified in this table. Numbers added in brackets refer the Michie’s BCT taxonomy (2013) or additional BCTs proposed by Abadje et al.*

To make delivery of the INTERPROF training as easy as possible, we developed several materials to share them with the participants both during the TTT and afterwards, including key SDM articles, useful links, the six steps to SDM pocket reminder cards, brochures and posters (samvalg.no), the INTERPROF presentation slides with explanatory texts (PowerPoint), the MAPPIN´SDM observer manual [33], several training videos demonstrating SDM consultations and several exercises including the use of barrier cards (Table 3). Those who complete the course qualify as “SDM ambassadors” and receive access to the log in page of the online platform hosting an even larger variety of materials and information (klarforsamvalg.no) [34].

**Table 3:** Learning objectives and content of the TTT training

	Learning objectives	Content	Communication format
Part one: Basic course (1 day)	**Knowledge** on background and rationale of SDM and risk communication **Skills** to structure an SDM process using “6 steps to SDM”	Demonstration of the SDM INTERPROF module background and description of SDM decisions relevant for SDM documented effects when SDM is used the SDM-process structured in six steps Criteria of risk communication	Lecture, practical video examples, group discussions
Part two: Advanced course (+ 2 days)	**Skills for teaching** SDM using **SDM INTERPROF** and for responding to typical trainee questions and concerns	Demonstration of the interactive part of SDM INTERPROF (using videos of clinical consultations) Prepared “barrier cards” are used in a facilitated discussion Demonstration of other learning resources on the klarforsamvalg.no	Lecture, group discussion, exercises
	**Competences** in **evaluating** SDM in consultations using quality criteria and in discussing quality of patient involvement	MAPPIN´SDM manual Appraisal of videos of HCP-patient consultations using the MAPPIN-observer scales	Edited training videos, observation exercises, demonstration of feedback provision, group discussions

The table gives an overview of learning objectives and corresponding content in the TTT and which communication form was used. The underpinning pedagogic approach is presented elsewhere [18]. BCTs applied in the TTT are indicated in detail in Table 2. INTERPROF refers to the corresponding SDM training module [19]. MAPPIN´SDM is a set of measurement scales assessing patient involvement in decision making [13, 14]

In an effort to achieve a balance between encouraging individual tailoring of the training while ensuring that SDM learning objectives are still met (fidelity), the curriculum prioritizes finding common ground in the concept of quality (MAPPIN’SDM). Special emphasis is, therefore, given to training in appraisal of the quality and extent of patient involvement in consultations. Observation- and appraisal exercises are used to teach participants to applying the criteria of the MAPPIN’SDM observer scales [13, 14].

Each single component of the TTT course (basic and advanced) had already been tested, either in other modules of the meta-curriculum or with the target group for the TTT, when we piloted the basic course with 40 HCPs in the South-Eastern Health Region. The piloting was followed by a four-hour focus group with about half of them. Based on their feedback and on experiences with single components of the TTT program, a three-day in-person workshop was considered as an appropriate format and time frame for achieving the learning objectives.

#### Description of the TTT

The basic course and the advanced course were organized as separate sessions to accommodate work schedules. Participants could sign up for the basic course and later decide to continue with the advanced course. Participation in the advanced course required completion of the basic course at any earlier date. However, both parts need to be completed to become an “SDM ambassador,” i.e. qualified to deliver the SDM INTERPROF training module.

##### Basis course Day 1

The first day (6 h) consists of an introduction (Table 3) and several exercises and group reflections, including on barriers to implementing SDM perceived by the trainers or the trainees and BCTs that could be used to overcome them (Table 2). Participants are introduced to the SDM-INTERPROF module and encouraged to particularly focus on the use of pedagogic methods within the training. After demonstration of the two-hour SDM INTERPROF module, the challenges of teaching it are discussed in depth. By attending the first day, participants are supposed to achieve similar knowledge about SDM as attendants of the INTERPROF module.

##### Advanced course Day 2–3

The second (6 h) and third day (6 h) consist of exercises in applying quality criteria through an in-depth analysis of patient involvement in decision-making. Videos of clinical consultations in several different domains are observed, analysed, and rated using the MAPPIN’SDM criteria, and then discussed at a group level. Alternating with sequences of the observer training are short lectures on SDM topics such as evidence-based patient information (EBPI) including risk communication or the stage of evaluation of the various modules of the Ready for SDM meta-curriculum as well as several exercises from the curriculum which participants will use in their training.

After the course, the participants receive a certificate of completion and are invited to join the quality collaborative of certified SDM ambassadors in the region. Ambassadors are introduced to the online platform [34] through which they can seek additional learning resources, improve existing resources, add BCTs, or suggest new resources which will pass thorough an appraisal process before approval by the originators (SK, JK) and being made accessible to the entire network.

### Setting and participants

**Fig. 1:**
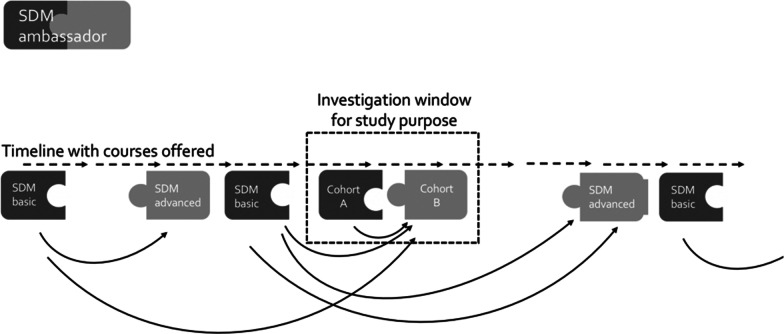
Recruitment of TTT participants

The study was conducted in the largest of Norway’s four regional health authorities which serves a population of 2.9 million people and has 11 health trusts. Recruitment was pursued through the Deputy CEO, the Chief Medical Officer and announcements posted on the South-Eastern Regional Health Authority’s website. Eligible applicants were either responsible for implementation of SDM in their local institutions or showed an interest in the course. The aim was to recruit a minimum of two HCPs from each health trust and to ensure the number of participants was under 25. Twenty-five is sufficient for conducting the intervention in a meaningful manner. Cohort A refers to trainers attending the basic course and cohort B to trainers participating in the advanced course. Some members of cohort B had pursued the basic course earlier, while others were also represented in cohort A, i.e. had moved directly from basic to advanced. Studying the same group through both parts of the TTT module instead of a composite sample would have been preferable but was not possible (see Fig. 1) for logistical reasons.

### Procedures

The intervention was delivered by SK and JK. SK is a registered nurse with a master’s degree in Health and Empowerment and is a PhD student focused on SDM training, as well as a special advisor for SDM at the South-Eastern Norway Regional Health Authority. JK is a psychologist, professor and communications researcher at the Oslo Metropolitan University. Both trainers have extensive experience in conducting SDM trainings.

Both parts of the TTT were held at the South-Eastern Norway Regional Health Authority meeting centre in Oslo in September 2019. Participants’ respective hospital trusts paid for their transportation costs and they attended within working hours.

Informed consent was signed in the context of the training session before handing out the questionnaires. Three months after the intervention, an online survey sent by email asked participants how many trainings they had offered and to how many HCPs. After one week, an email reminder was sent out. A follow up workshop was planned with the participants after six months for evaluation purposes and to accompany the HCP in their implementation efforts.

### Outcome measures

Cohort A was given a self-administered paper questionnaire before they left the basic course on Day 1, and Cohort B were given a self-administered paper questionnaire before leaving the advanced course at the end of Day 3. Cohort B then completed outcome measures at 3-months follow up (Table 5). Demographic characteristics were collected. Outcomes were related to the three Kirkpatrick levels of reactions, learning, and behaviour. Nine items evaluated reaction outcomes (Kirkpatrick level 1—engagement, relevance and satisfaction). Twenty-nine items evaluated learning outcomes (Kirkpatrick level 2—knowledge, attitude, skills, confidence, commitment) (Table 5) [27]. Twelve of the items used 4-point Likert scales (1 = strongly disagree, 4 = strongly agree). Five items on knowledge were assessed using multiple choice (definition of SDM, indications and contra-indications, prerequisites for informed choice, reliable sources of information about effects of medical interventions) [18]. These items have been piloted and are in use for certifying graduates of an SDM e-tutorial [35]. They have also been used as one of two endpoints in the corresponding cluster randomized trial, evaluating the INTERPROF module [24]. By measuring SDM related knowledge of participants the current study intended to assure that attendees of the TTT would acquire knowledge not inferior to the attendees of the INTERPROF training. Level 2 skills outcomes were additionally measured at the end of Day 3 when participants were asked to assess patient involvement in decision-making within a test video of a consultation, using the observer-based instrument MAPPIN-O_dyad_ [15, 16]. The latter assesses the dyad's (HCP and patient as a unit) compliance with 11 quality criteria of patient involvement in a decision-making consultation [15, 16]. The criteria are rated from ‘0′ (not observed) to ‘4′ (observed to an excellent standard) [15, 16]. Finally, for the behaviour outcomes (Level 3), three months after the TTT-courses, an online survey was sent to all those who completed both courses to assess the number of SDM trainings carried out and the number of HCPs trained. Additionally, open-ended questions were used to collect implementation outcomes such as relevance (applicability), satisfaction (need for revision) and barriers to conducting the trainings (Table 5).

### Statistical analyses

#### Data administration

All quantitative data were entered into SPSS version 22.0 (IBM corporation, USA). The qualitative data from items using open-ended questions were entered into NVivo version 11 (QSR International, Melbourne, Australia).

#### Analysis of quantitative data

Data from the post intervention paper questionnaire were calculated using frequencies and either reported as percentages of the answer categories (Engagement, Relevance, Satisfaction, Knowledge, Attitude, Confidence, Commitment, Age interval, Years of clinical practice) or, if continuously scaled, averaged and reported as mean scores (Knowledge). Levels of knowledge after the basic course were compared with knowledge levels in the intervention group of the corresponding RCT evaluating the INTERPROF module and tested for noninferiority using a one-sided t-test [24]. Missing values were reported separately.

#### Analysis of qualitative data

Data collected using open-ended questions were analysed based on principles of qualitative content analysis as described by Hsieh and Shannon [36]. Data extraction and analysis were undertaken by two independently working researchers using the following steps: (a) reading the answers multiple times to establish a sense of the data as a whole, and to identify meaningful units; (b) categorizing units based on a priori defined main themes (Kirkpatrick’s levels) and subcategories; and (c) resolving disagreements through discussion at each step described above.

#### Analysis of observer skills

Accuracy of participants’ appraisals of SDM behaviours, collected with the MAPPIN´SDM dyad observer scale, was determined by calculating the extent to which ratings agreed with a reference standard. The reference standard was a rating established by experts in MAPPIN’SDM before the study started. Agreement was expressed using the weighted t coefficient [37], a Cohens kappa, modified according to Maxwell [38] that uses theoretical assumptions rather than empirical frequencies to estimate the expected marginal distributions. Agreements were downgraded from full agreement (= 1), over almost (1 Likert step = 0.75), moderate (2 Likert steps = 0.25), low (3 Likert steps = 0.1) to no agreement (4 Likert steps = 0). Coefficients are considered moderate between 0.40 and 0.60, strong higher than 0.60, and excellent higher than 0.80 [39].

## Results

Results of the study are reported following Kirkpatrick’s first three evaluation levels.

### Characteristics of training participants

Nine of the 11 hospital trusts sent participants to the TTT course. Six of them sent more than one participant. Written informed consent was provided by 21 of 24 participants in Cohort A and 19 of 22 in Cohort B (Table 4).

**Table 4:** Characteristics of participants in a train-the-trainer program for SDM

	Cohort A N = 21 (%)	Cohort B N = 19 (%)
*Sex*		
Female	15 (71)	14 (74)
Male	6 (29)	5 (26)
*Age*		
30–50 years	10 (48)	7 (37)
> 50 years	11 (52)	12 (63)
*Profession*		
Registered Nurses	9 (43)	8 (42)
Physicians	5 (24)	0 (0)
Advisors/Special Advisors /Leaders	6 (28)	7 (37)
Physiotherapists	1 (5)	2 (10.5)
Social Educators	1 (5)	1 (5)
Occupational Therapists	1 (5)	2 (10.5)
Reported mixed positions	4 (19)	1 (5)
*Position*		
Professional development and teaching	11 (48)	12 (63)
Management and administration	9 (43)	10 (52)
Clinical practice	7 (35)	2 (10.5)
Reported mixed positions	6 (29)	6 (33)
*Years of clinical practice*		
Over 6 years	20 (95)	15 (83)

### Level 1: reaction

#### Engagement

The TTT program was evaluated as interesting by 95% of participants (19 of 20) after the basic course and by 74% (14 of 19) after the advanced course. After the basic course, participants said technical problems may have interfered with learning. The wish for less ambitious materials and for a gold standard SDM video were expressed after the advanced course (Table 5).

**Table 5:** Overview over the entire evaluation plan and corresponding results

Outcome	Operationalization		Time	Results
*Level 1: reactions*				
Engagement	Interest: *The workshop kept your interest throughout the day (s)*	Self-reported, questionnaire (4-point Likert scale)*	After basic	95% (19 of 20) agreed or strongly agreed
			After advanced	74% (14 of 19) agreed or strongly agreed
	Interferences: *Was there anything about the course that interfered with my learning process?*	Self-reported questionnaire (open-ended question)	After basic	*Challenges regarding technique issues and the room where the course was held* *We would wish to see a SDM gold standard video*
			After advanced	*A bit theoretical* *I was frustrated since I felt low agreement in the observer training* *The MAPPIN´SDM manual should be simplified in terms of language and examples* *Too few breaks*
Relevance	Usefulness & applicability *I consider what I learned through the workshop as useful for carrying out my work*	Self-reported questionnaire (4-point Likert scale)*	After basic	90.5% (19/21) agreed or strongly agreed
	*Through the workshop I became aware of how I can apply what I have learned*			86% (18/21) agreed or strongly agreed
	Usefulness & applicability *I consider what I learned through the workshop as useful for carrying out my work*		After advanced	68% (13/19) agreed or strongly agreed
	*Through the workshop I became aware of how I can apply what I have learned*			63% (12 of 19) agreed or strongly agreed
	Applicability*:* *What elements in the TTT course have been most useful for you?*	Self-reported, online questionnaire (open question)	After three months	*Watching video clips and recognizing the steps to SDM* *Learning about how to disseminate the steps of an SDM process* *Day one was absolutely most helpful. Both to gain basic knowledge about SDM, what, why, how. Very good with all the teaching material that was ready for use* *Learning about the difference between this and other communication methods with patients regarding health decisions* *To watch videos where SDM is actually happening and to understand that SDM is a sequence in a patient conversation* *Interprofessional discussions of different perspectives within the group* *Interesting to watch videos in light of the 6 steps of an SDM process, but also challenging* *Becoming well acquainted with the purpose and understanding behind SDM* *Day two did not quite meet what I needed in terms of conducting teaching in practice. Nice to see and reflect on videos, but a bit too extensive and deep-diving*
Satisfaction	Willingness to recommend: *I would recommend this course to others who are going to provide SDM training*	Self-reported questionnaire (4-point Likert scale)*	After basic	95% (20 of 21) agreed or strongly agreed
			After advanced	56% (11 av 19) agreed or strongly agreed
	Need for revision*:* *What can we do to improve the TTT-course?*	Self-reported, online questionnaire (open ended question)	After three months	*Frame it less research oriented* *The course was good, good lectures and instructive content on lecture. Nice balance between exercises / assessment of videos / self-activity and lecture* *Clarify the relationship between “four habits”* [39, 40]*, “choosing wisely”* [38] *and the Norwegian campaign “what is important for you”* *Put more focus on anchoring within the hospital trust and strategic work on implementing SDM* *Fewer videos to allow for more in-depth review and professional discussion* *More practice in how to perform the training* *More practicing in how to assemble the SDM training based on different needs and more training in didactics* *More time for discussing experiences with teaching* *More time for discussing how to reach the various target groups*
*Level 2: learning*				
Knowledge	Subjective understanding SDM: *I have understood the concept of SDM*	Self-reported questionnaire (4-point Likert scale)*	After basic	95% (20 of 21) agreed or strongly agreed
	Subjective understanding SDM: *I know the quality criteria for SDM*		After advanced	84% (16 of 19) agreed or strongly agreed
	Five-item SDM knowledge test SDM is indicated Patient involvement in decision-making means What does the patient need to make an informed choice? Which knowledge base is used to consider the benefit of medical interventions? When is SDM contraindicated?	Multiple-choice questions	After basic	Participants knowledge scored higher than knowledge measured in the training group in an earlier RCT [24]
Attitude	Attitudes regarding patient involvement *It is desirable to involve patients in medical decisions*	Self-reported questionnaire (4-point Likert scale)*	After basic	100% (21 of 21) agreed or strongly agreed
			After advanced	100% (19 of 19) agreed or strongly agreed
	*It is beneficial for health care providers to receive SDM training*			95% (21 of 21) agreed or strongly agreed
	Attitudes regarding the INTERPROF module: *It is beneficial for health care providers to receive SDM INTERPROF training*			100% (19 of 19) agreed or strongly agreed
Skills	Accuracy of communication judgements	Observation test using MAPPIN	After advanced	Participants attained excellent skills in quality appraisal (mean = .80, N = 19)
Confidence	Regrading handling barriers, conducting training, conveying SDM appraisal: *I am able to convey what SDM means to others*	Self-reported questionnaire (4-point Likert scale)*	After basic	81% (17 of 21) agreed or strongly agreed
	*I am able to answer typical questions / barriers about SDM*			86% (18 of 21) agreed or strongly agreed
	*I am able to assess degree/extent of SDM within HCP-patient consultations*		After advanced	68% (13 of 19) agreed or strongly agreed
	*I can convey to what extent / why / why not SDM is being conducted*			53% (9 av 17) agreed or strongly agreed
	*I feel confident in providing SDM INTERPROF training to HCP*			53% (10 of 19) agreed or strongly agreed
	Confidence to conduct training: *What additional support would you need to complete 2-h training?*	Self-reported questionnaire (open ended questions)		*More practice exchange with other SDM trainers* *More information about adult learning methods* *More exercise in assembling components of the curriculum to tailor the SDM training to the target audiences* *More practical training and experience* *Guidance from the course developers later on* *More training resources, clinical videos on klarforsamvalg.no* *More observer training* *Practical advice on how to get clinicians to set aside time / prioritize*
	Confidence to conduct training: *What are the barriers to delivering SDM trainings?*			*Lack of time of the target group* *Uncertainty regarding how to invite and get access to clinicians* *Anchoring SDM in the management at the hospital* *Feeling not yet confident enough to provide skills-training using clinical videos with feedback provision and group discussions rather than just delivering information about SDM*
Commitment	Concrete plans for realization: *I consider it to be likely that I will teach others (HCP) in SDM within the next 6 months*	Self-reported questionnaire 4-point Likert*	After advanced	42% (8 of 19) agreed or strongly agreed
*Level 3: Behaviour*				
Realization	Number of trainings performed; number of HCPs trained	Self-reported, online questionnaire	After three months	69% (9 of 13) had conducted SDM trainings 62% (8 of 13) had conducted more than two SDM trainings 458 HCPs had received training
Barriers	Preparation: *It was easy to construct a training using the materials provided on the platform*	Self-reported, online questionnaire (4-point Likert scale)*		64% (6/11) agreed or strongly agreed
	Barriers met during training conducts: *What were the barriers to delivering the SDM trainings?*	Self-reported, online questionnaire (open ended questions)		*Lack of opportunities to deliver the trainings* *Difficult access to clinicians caused by time limitations and lack of invitation* *The absence of an implementation strategy for the hospital trust* *Challenging to know the program and to adapt it, even though the resources on the webpage were very helpful* *It is a difficult role to convey with credibility and dedication if you yourself are no longer working clinically* *The SDM presentation might be considered very theoretical and even as another “must” task. HCP feel they are doing SDM already*

This table presents an overview over the entire evaluation plan and corresponding results structured according to Kirkpatrick’s levels of evaluation. In the study, all questions were provided in Norwegian language. * the 4-point Likert scale ranged from 1 = “strongly disagree” to 4 = “strongly agree”

#### Relevance

After the basic course, 90.5% of participants (19 of 21) considered the course helpful for their job and 68% (13 of 19) after the advanced course. After the basic course 86% (18 of 21) said that they learned how they could apply their new skills and 63% (12 of 19) after the advanced course.

Three months after the course, participants said they had a better understanding of the difference between SDM and other communication concepts, of the six steps to SDM, of SDM as part of a broader communication approach, and of how to recognize the SDM steps (through having watched and analysed the SDM consultation videos).

#### Satisfaction

Most TTT participants (95%) (20 of 21) would recommend the basic course to colleagues, while 56% would recommend the advanced course (11 of 19).

#### Suggestions for improving the 3-day TTT

Respondents suggested: (1) More practice in how to perform the training, (2) more focus on embedding SDM within the local hospital trusts, and (3) strategic work regarding SDM implementation. Additionally, participants desired more time for in-depth analyses and discussion of clinical consultations. They were also confused between communication campaigns such as Choosing Wisely [43], Four Habits [44, 45] and Ready for SDM. They also requested theoretical and pedagogical background and exercises on how to assemble an SDM course tailored to local needs.

### Level 2: learning

#### Knowledge

95% (20 of 21) of the TTT participants considered the concept of SDM and 84% (16 of 19) the Patient involvement in decision-making indicators understandable.

Knowledge levels acquired during TTT were not inferior to knowledge acquired by course participants in the RCT, both for each single item on the knowledge test and for the mean score (mean training group RCT = 2.9, (range 0–5), SD = 1.40, TTT = 4.28, (range 0–5), SD = 0.72; p < 0.001).

#### Attitudes

After the basic and the advanced courses, all participants held positive attitudes towards SDM in general and towards training HCPs in SDM. Additionally, all participants considered it valuable to use videos of clinical consultations in combination with quality criteria for SDM in the training.

#### Skills

After the three-day TTT, skills in observing and assessing communication quality in terms of MAPPIN-O_dyad_ were excellent. According to weighted t, participants’ assessment of SDM behaviour presented in the test video agreed to a high extent with the reference standard (mean of weighted t = 0.80, N = 19).

#### Confidence

After finishing the TTT, participants felt confident to handle typical questions about and barriers to SDM (86%, 18/21) and to convey the meaning of SDM to others (81%, 17/21). However, self-confidence after the course was lower with regard to assessing patient involvement in making decisions using the quality criteria of the MAPPIN’SDM (68%, 13/19), to justifying and communicating their appraisal to others (53% / 9 of 17) and to conduct the 2-h SDM training with a group of HCPs (53% /10 of 19).

#### Commitment

Forty-two percent of the participants (8/19) left the course with concrete plans to conduct 2-h SDM trainings within the next six months.

#### Other feedback on learning

The participants wanted more interprofessional examples, more self-study, and more time for going through the teaching materials. They asked for more exercises in assembling components of the SDM INTERPROF curriculum to tailor the SDM training to their target audiences, more practical training and experience, more guidance from the course developers, and more training materials such as the clinical videos on the online platform klarforsamvalg.no [34].

### Level 3: Behaviour

#### Realization of SDM training

Three months after TTT, 85% (11/13) of the participants still available for evaluation had been given a dedicated task from their leaders at the hospital trust to deliver SDM trainings, of whom 69% (9) had carried out SDM trainings and 62% (8) more than two trainings. In total, 458 HCPs had received training up to this point (Table 5).

#### Implementation issues

Barriers to conducting SDM trainings reported immediately after training were largely similar to barriers reported three months later. These were lack of time, limited access to clinicians for training, insufficient support from leaders, the complexity of the training, and insufficient self-confidence. Participants wanted more training in giving information about SDM and in providing video supported skills-training. Some trainers struggled (4/11) to adapt the training to the local culture and to their own needs and situation. Six of 11 (64%) survey respondents considered it easy to organize their SDM training programs using the material available on the platform.

## Discussion and conclusion

### Discussion

The current study evaluated a TTT for HCPs to prepare them to conduct a 2-h SDM INTERPROF training previously proven feasible and efficacious for changing SDM-related competencies [18, 24]. The TTT program uses a “blended learning” approach [40] that combines didactic and interactive techniques and learning materials.

While the one-day basic course was positively evaluated by the participants, the three-day advanced course received a more variable response (e.g., acceptability) and self-assessed outcomes (e.g., confidence). On the other hand, most participants were committed to conducting training sessions in the future and 69% did so. Almost two-thirds of those completing the advanced course carried out more than two trainings each within three months, and 458 HCPs were trained in total. In terms of their ability to observe behaviour regarding patient involvement and perform reliable quality appraisal of SDM participants scored well. Also, knowledge gained was high (range: 71–100%), and even superior to knowledge levels acquired by a comparable group of participants in the RCT (range: 41%-83%) [24] (Table 5).

Our study has several key limitations. As many outcomes were self-reported, our findings might be biased due to social desirability. This risk, however, mainly applies to participants’ assessments of relevance, satisfaction and subjective knowledge, while other outcomes, like numbers of provided training sessions, are unlikely to be overestimated. Our evaluation is also lacking continuity with regard to ambassadors and feedback provided by them, because it was based on a composite assessment of two cohorts moving separately through the two parts of the curriculum. In the absence of closed groups passing the entire program, we had to choose this proceeding to ensure data collection in a limited time frame. About half of the participants, however, did indeed continue from the basic to the advanced course in our investigation window and were therefore present in both cohorts. We do not see any reason to believe that heterogeneity between the cohorts caused undesirable variance. As the current study was not designed to answer questions about efficacy in terms of patient-relevant outcomes the fourth Kirkpatrick level has not been addressed. However, evaluations covering the entire spectrum are rare. Assessing the program’s impact on patient involvement in decision-making will be of particular importance in the broader context of the literature on SDM trainings [41] and TTT in general [42–45].

Evaluating trainers’ (ambassadors) learning outcomes and behaviours (rather than the trainee/patient variables) is a unique [40] although reasonable strategy, as suggested by a recent review [42] and the Kirkpatrick Model [27]. As part of the development process [24], this approach may lead to a better understanding about the best way to support trainers in training others, and a better understanding of the development of trainers’ learning abilities and behaviour over time [41]. Our recruitment strategy resulted in a mix of participants who had been sent by their hospital trusts and participants who came of their own volition. While the latter group could have caused selection bias, interestingly, we observed that participants commissioned by their leaders and those motivated by interest only were equally as likely to perform further trainings. Finally, the three-month follow up may have been too soon. We chose this time frame because of how long it takes to plan and hold meetings in the context of the Norwegian specialist health care. Moreover, we assumed that participants would not retain their new skills unless they put them to use, and hoped the three-month evaluation would motivate them to quickly apply their knowledge and skills [27]. We also assumed participants who started training HCPs soon after the TTT would be more likely to continue later on.

We learned from the current study that we have still not found the best blend of learning techniques, particularly for the advanced course focusing on SDM observer skills. This challenge is found elsewhere in the literature on dissemination strategies for TTT programs [40]. A systematic review of 18 TTT programs for health and social care professionals is inconclusive regarding the optimal blend, but in general recommends variation between didactics and interactive teaching methods. Our findings reveal the need to further analyse barriers and include further BCTs, for example, offering exercises on preparing, tailoring and piloting SDM lectures and providing feedback before the trainers conduct them in their hospital trust. Putting the original course into practice without any variation is an illusion but participants did not feel adequately equipped to tailor the course to their context. Future TTTs could identify key SDM concepts that require fidelity but also support and encourage participants to reflect on and adapt the training to their own context and develop their own style of training. Their confidence in giving the course could be increased by strengthening their teaching skills, practicing individual and group work, providing a simpler version of the MAPPIN’SDM materials (coding scheme and manual) and revising the webpage that houses the teaching materials. In addition, participants expressed frustration regarding the observer training. Having in mind the excellent accuracy findings, we do however think, that providing more immediate feedback on their observer skills (BCT: Feedback on behaviour (2.2))’ could have improved their motivation and self-efficacy.

The Ready for SDM program is innovative in at least four respects: Firstly, it is the first and only evaluated SDM training program in Norway [5, 6]. Secondly, it is based on a meta-curriculum which provides a variety of training components tailored to various contexts and HCPs. Thirdly, to the best of our knowledge, the Ready for SDM program is the first of its kind to provide evaluated TTT methods. There is only one other evaluated TTT program in the field of SDM [46], but none in Norway [5, 41, 42]. Fourthly, in order to resolve the challenge of maintaining training quality when passing it along, Ready for SDM certifies trainers in an SDM training quality collaborative [47]. This growing network meets both in workshops and on the online platform that stores the training materials. Using a feedback-driven continuous learning system, Ready for SDM enables members to participate in program revisions and further development and limiting the proliferation of training programs that do not meet quality standards. Feedback-driven continuous learning systems are well-known in developmental evaluation research [48, 49], and are assumed to give trainers enough flexibility to develop further skills and a sense of ownership of the methods they are supposed to apply. Other studies are needed to evaluate whether this approach also has an impact on sustainability.

Since our study, most SDM trainers in the quality network have continued to spread knowledge and a positive attitude towards SDM while delivering the SDM INTERPROF trainings within the South-Eastern Health Region. The program now needs to be considered in the context of a broader research agenda for the Ready for SDM meta-curriculum and the even broader agenda of a national implementation plan for the Norwegian health care system. Through the architecture of the feedback-driven continuous learning system of the meta-curriculum and its evaluation concept, Ready for SDM is resolving the challenges reported in the literature [5, 6, 50] such as lack of transparency regarding content and methods, inappropriateness of evaluation methods and the fact that most programs are targeting only doctors. It is also working towards a regional consensus on what constitutes fidelity, i.e. which elements of training content are essential and which can be tailored or replaced. While the curriculum is still under development and rigorous proof of efficacy and effectiveness of all modules will need more time. Ready for SDM offers a comprehensive implementation approach that includes all players in the processes of health communication and decision-making. This approach is based on evidence suggesting that a combination of strategies targeting patients, HCPs and structural changes to promote patient involvement [3, 4, 51–54] will be most effective.

### Conclusion

Our study showed that training SDM ambassadors to provide the SDM INTERPROF module helped scale up SDM training activities in the hospital trusts. The TTT improved knowledge and produced excellent observer skills in assessing patient involvement in decisions. However, some trainers felt insufficiently confident to perform further trainings and to convey the concept of quality of patient involvement in decision-making to other HCPs. Ambassadors provided rich feedback which will inform the revision of the TTT program. Further research is required regarding efficacy of the TTT in the context of a comprehensive multifaceted strategy for implementing SDM in clinical practice countrywide.

## Supplementary Information

**Additional file 1.** Five-item knowledge test.

**Additional file 2.** TIDieR checklist.

**Additional file 3.** MAPPIN'SDM coding sheet.

## Data Availability

The datasets used and/or analysed during the current study are available from the corresponding author upon reasonable request.

## Abbreviations

TTT: Train-the-trainer
SDM: Shared decision-making
HCP: Health-care provider
BCTs: Behaviour change techniques
MAPPIN’SDM: Multifocal approach to sharing in shared decision making
KTA: Knowledge-to-action framework
EBPI: Evidence-based patient information
