# Supplementary material for: Ready for SDM: evaluating a train-the-trainer program to facilitate implementation of SDM training in Norway
Source: BMC Med Inform Decis Mak. 2021 Apr 30;21:140. doi: 10.1186/s12911-021-01494-x (PMC8086335; doi:10.1186/s12911-021-01494-x)
Supplement: Supplementary file 1 — Additional file 1. Five-item knowledge test. [file 12911_2021_1494_MOESM1_ESM.docx]

**Five-item multiple-choice knowledge tests**

(Freely translated from the Norwegian version)

**SDM is indicated**

1. Always when the patient wants to negotiate.

2. Only if the options are equally effective.

3. Only if the patient explicitly wishes to participate in the decision.

**4. Always if there are several options, and if the patient is conscious.**

5. Always, when from a medical perspective, there is only one option.

**Patient involvement in decision making means**

1. That decisions are made in line with scientific evidence.

2. That the patient may wish prefer a specific option.

**3. That the patient is informed about all the options and is supported in making a decision.**

4. That the HCP does not make recommendations.

5. That the HCP takes the patient's preferences into account when making a decision.

**What does the patient need to make an informed choice?**

1. A detailed understanding of the physiology of the target organ of the intervention.

2. Numerical data on benefits and harms of each alternative, even if drawn from cohort studies.

3. Certainty, even if this is not supported by scientific evidence.

4. Prevalence of the disease in the country where the patient lives.

**5. Quantification of benefits and harms of each alternative, only if evidence based.**

**Which knowledge base is used to consider the benefit of medical interventions?**

1. Comprehensive documented case studies

**2. Randomized controlled trials**

3. Cohort studies

4. The clinicians’ own heuristics drawn from their clinical experiences.

5. Epidemiological studies

**When is SDM contraindicated?**

**1**) When it is uncertain whether the patient would understand the information.

2) When scientific evidence exists indicating a benefit for one of the possible interventions.

3) If the patient clearly isn’t motivated to form his/her own opinion.

**4) When there is only one option available.**

5) When the guideline clearly recommends one of the alternatives.
