# Supplementary material for: Ready for SDM: evaluating a train-the-trainer program to facilitate implementation of SDM training in Norway
Source: BMC Med Inform Decis Mak. 2021 Apr 30;21:140. doi: 10.1186/s12911-021-01494-x (PMC8086335; doi:10.1186/s12911-021-01494-x)
Supplement: Supplementary file 2 — Additional file 2. TIDieR checklist. [file 12911_2021_1494_MOESM2_ESM.docx]

**
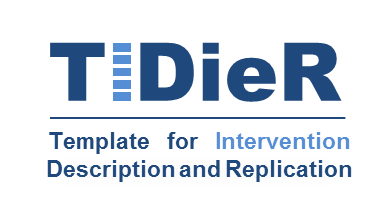
 The TIDieR (Template for Intervention Description and Replication) Checklist*:**

Information to include when describing an intervention and the location of the information

| 1. **BRIEF NAME**: | **Where located** |
| --- | --- |
| 1. INTERVENTION: Ready for SDM - Train-The-Trainer program to facilitate implementation of SDM training in Norway. | Page 1. |
| 1. **WHY** |  |
| THEORY: As the TTT is an implementation strategy, its development was done in accord with the Knowledge-to-Action framework (KTA).  LEARNING OBJECTIVES : The main learning objective of the TTT course is to build competence and confidence among SDM “ambassadors” who will provide the Ready for SDM INTERPROF module to further groups of HCPs.. | Page 6-7.  Page 9. Table 3. |
| 1. **WHAT** |  |
| **MATERIALS:** The Ready for SDM Train the trainer was designed to facilitate implementation of SDM training in Norway. See Table 3: Learning objectives and content of the interprofessional TTT module for SDM training for more information about materials used. Additional information can be found on the projects webpage at klarforsamvalg.no  **EDUCATIONAL STRATEGIES:** Using “blended learning” and adult learning approaches (31) as well as strategies from the Ready for SDM meta-curriculum (18, 21), the TTT includes presentations, group discussions, exercises, interactive observation and demonstration**.** Pedagogic methods were selected that were appropriate for bigger groups and still keeping focus on interactivity in the learning. The course was designed to help trainers address known barriers to SDM .  **INCENTIVES:** Participants were not paid and there were otherwise no financial incentives. | Page 9. Table.  Page 9. |
| 1. **WHO PROVIDED** |  |
| **INSTRUCTORS:** The intervention was delivered by SK and JK. SK is a registered nurse with a master’s degree in Health and Empowerment and is a PhD student focused on SDM training, as well as a special advisor for SDM at the South-Eastern Norway Regional Health authority. JK is a psychologist, professor and communications researcher. Both trainers have extensive experience in conducting SDM trainings. | Page 13-14. |
| 1. **HOW** |  |
| DELIVERY: A three days’ face to face workshop was considered as an appropriate format and timeframe to achieve the learning objectives based on previous experiences piloting single components of the TTT program | Page 11. |
| 1. **WHERE** |  |
| ENVIRONMENT: Both parts of the TTT were held at the South-Eastern Norway Regional Health Authority meeting centre in Oslo in September 2019. Participants respective Hospital Trusts paid for the transport to the venue. | Page 14. |
| **WHEN and HOW MUCH** |  |
| 11. SCHEDULE:  Part one: basic course:   - Dissemination of learning goals - Conditions - SDM Basics - When is the SDM teaching relevant? - The SDM INTERPROF teaching package - Interactive training - Dealing with typical barriers and questions   Part two: advanced course:   - Update since part one - Presentation of SDM INTERPROF interativ training - About the MAPPIN SDM - The goal of the observer training - Observer Training (View and rate consultations with MAPPIN code sheets) - Discussions of the extent and quality of SDM in the consultations - How to communicate the essence of the MAPPIN analysis for in the SDM INTERPROF training - short lectures on different topics (e.g. on evidence-based patient information (EBPI), risk communication) - Different exercises (e.g., with case vignettes) - A trained SDM ambassador – what now?   The basic course lasted for 6 hours. The advanced course for additional 2 days (2x 6 hours). | Page 12. |
| **TAILORING** |  |
| The intervention was tailored to the target group based on previous piloting and educational strategies. | Page 7-8. |
| **MODIFICATIONS** |  |
| The target group was involved in the development of the TTT in a quality improvement project. After that some minor changes were made before conducting the current study.  There were no unplanned changes during the course. | Page 10. |
| **HOW WELL** |  |
| ATTENDANCE: All participants attended both parts of the course (as planned). |  |

Hoffmann TC, Glasziou PP, Boutron I, et al. Better reporting of interventions: template for intervention description and replication (TIDieR) checklist and guide. BMJ 2014; 348: g1687.
