# Supplementary material for: Ready for SDM: evaluating a train-the-trainer program to facilitate implementation of SDM training in Norway
Source: BMC Med Inform Decis Mak. 2021 Apr 30;21:140. doi: 10.1186/s12911-021-01494-x (PMC8086335; doi:10.1186/s12911-021-01494-x)
Supplement: Supplementary file 3 — Additional file 3. MAPPIN'SDM coding sheet. [file 12911_2021_1494_MOESM3_ESM.docx]

| **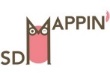** | | **MAPPIN`SDM – coding sheet** | | | | | | | |
| --- | --- | --- | --- | --- | --- | --- | --- | --- | --- |
|  |  | Below is a list of quality criteria used to evaluate medical consultations regarding whether the patient was involved in decisions concerning his / her own health. Please go through and enter your own assessments of the conversation by giving the statements below a score between 0 and 4. | | | | | | | |
| **Use the following ratings** | | |  | |  | |  |  |  |
|  | |  | **0** | **=** | | **«The behaviour is not observed»** | | | |
|  | |  | **1** | **=** | | **«The behaviour is observed (minimal effort)»** | | | |
|  |  |  | **2** | **=** | | **«The basic competence has been observed »** | | | |
|  |  |  | **3** | **=** | | **«The behaviour is observed (goud standard)»** | | | |
|  |  |  | **4** | **=** | | **«The behaviour is observed to an excellent standard»** | | | |

| **1**  **Defining problem** | Clinician and patient agree on a concrete problem as one that requires a decision-making process. | **Score 0-4:** |
| --- | --- | --- |
|  |  |  |

| **2**  **SDM- key message** | Clinician and patient discuss that there is more than one way to deal with the concrete problem (SDM key message). | **Score 0-4:** |
| --- | --- | --- |
|  |  |  |

| **3a**  **Options (quality of the structure)** | Clinician and patient structure the discussion of the options in a way that is easy to understand and easy to remember. | **Score 0-4:** |
| --- | --- | --- |
|  |  |  |

| **3b**  **Options (quality of the content)** | Clinician and patient weigh up the pros and cons of the different options (if applicable, also the pros and cons of ‘doing nothing’). | **Score 0-4:** |
| --- | --- | --- |
|  |  |  |

| **3c**  **Options (information quality)** | Clinician and patient consider the criteria of evidence based patient information (presentation, sources, level of evidence). | **Score 0-4:** |
| --- | --- | --- |
|  |  |  |

| **4**  **Expectations & worries** | Clinician and patient discuss the patient’s expectations (ideas), concerns (fears) and preferences about how to manage the concrete problem. | **Score 0-4:** |
| --- | --- | --- |
|  |  |  |
|  | | |
| **5**  **Indicate decision** | Clinician or patient opens the decision stage leading to the selection of an option (If applicable, deferment is a possible decision). | **Score 0-4:** |
|  |  |  |

| **6**  **Follow up arrangements** | Clinician and patient discuss plans for how to proceed (e.g. steps for implementing the decision, review of decision or of deferment). | **Score 0-4:** |
| --- | --- | --- |
|  |  |  |

| **7**  **Communication** | Clinician and patient choose an approach to exchanging information (setting, media, time frame). | **Score 0-4:** |
| --- | --- | --- |
|  |  |  |

| **8**  **Evaluation of patient’s understanding** | Clinician and patient clarify whether the patient understood the information given by the clinician correctly. | **Score 0-4:** |
| --- | --- | --- |
|  |  |  |

| **9**  **Evaluation of doctor’s understanding** | Clinician and patient clarify whether the clinician has understood the patients viewpoint correctly. | **Score 0-4:** |
| --- | --- | --- |
|  |  |  |
